# Versatility in phospho-dependent molecular recognition of the XRCC1 and XRCC4 DNA-damage scaffolds by aprataxin-family FHA domains

**DOI:** 10.1016/j.dnarep.2015.10.002

**Published:** 2015-11

**Authors:** Amy L. Cherry, Timothy J. Nott, Geoffrey Kelly, Stuart L. Rulten, Keith W. Caldecott, Stephen J. Smerdon

**Affiliations:** a Francis Crick Institute, Mill Hill Laboratory, The Ridgeway, Mill Hill, London NW7 1AA, UK; b MRC Genome Damage and Stability Centre, University of Sussex, Brighton BN1 9RQ, UK

**Keywords:** APLF, aprataxin- and PNK-like factor, PNKP, polynucleotide kinase phosphatase, FHA, forkhead-associated, XRCC1, X-ray repair cross-complementing protein 1, XRCC4, X-ray repair cross-complementing protein 4, PARP-1, poly(ADP-ribose)polymerase 1, NHEJ, non-homologous end-joining, DNA-PKcs, catalytic subunit of DNA-dependent protein kinase, CK2, casein kinase 2, HIT, Histidine triad, ZF, zinc finger, NTD, N-terminal domain, BRCT, BRCA1 C-terminal, CC, coiled-coil, ITC, isothermal titration calorimetry, APTX, aprataxin, FHA domain, DNA break repair, DNA-damage signalling

## Abstract

Aprataxin, aprataxin and PNKP-like factor (APLF) and polynucleotide kinase phosphatase (PNKP) are key DNA-repair proteins with diverse functions but which all contain a homologous forkhead-associated (FHA) domain. Their primary binding targets are casein kinase 2-phosphorylated forms of the XRCC1 and XRCC4 scaffold molecules which respectively coordinate single-stranded and double-stranded DNA break repair pathways. Here, we present the high-resolution X-ray structure of a complex of phosphorylated XRCC4 with APLF, the most divergent of the three FHA domain family members. This, combined with NMR and biochemical analysis of aprataxin and APLF binding to singly and multiply-phosphorylated forms of XRCC1 and XRCC4, and comparison with PNKP reveals a pattern of distinct but overlapping binding specificities that are differentially modulated by multi-site phosphorylation. Together, our data illuminate important differences between activities of the three phospho-binding domains, in spite of a close evolutionary relationship between them.

## Highlights

• Aprataxin and APLF FHA domains show distinct specificities for XRCC4 and XRCC1.
• Specificity is modulated by multi-site phosphorylation.
• The crystal structure of APLF FHA and triphosphorylated XRCC4 peptide reveals a conserved mode of core pSpT motif binding.
• Overall phospho-dependent binding versatility of Aptx-family FHA domain proteins is provided by FHA surface basicity.

## 1 Introduction

Genome integrity is under constant threat from a variety of endogenous and exogenous genotoxic agents, which create a broad spectrum of both single-stranded and double-stranded DNA lesions. Failure to repair such breaks can result in cell death or tumor development. Several complex repair pathways have evolved to resolve various types of DNA lesions; these involve stages of break detection, DNA end-processing, DNA gap filling and DNA ligation. XRCC1 is a crucial scaffolding protein in base excision repair that interacts with and coordinates many known components of that pathway. These include poly(ADP-ribose) polymerase 1 (PARP-1) [1], [2], polynucleotide kinase phosphatase (PNKP) [3], aprataxin [4], [5], [6], [7], [8], aprataxin- and PNKP-like factor (APLF) [9], [10], DNA polymerase β [1], [11] and DNA ligase IIIα [12], [13]. XRCC4 has a parallel role in the non-homologous end-joining (NHEJ) pathway for repair of double-stranded DNA breaks where it interacts with the catalytic subunit of DNA-dependent protein kinase (DNA-PK_cs_) [14], [15], PNKP [16], aprataxin [4], APLF [17], [18] and DNA ligase IV [19], [20].

**Fig. 1:**
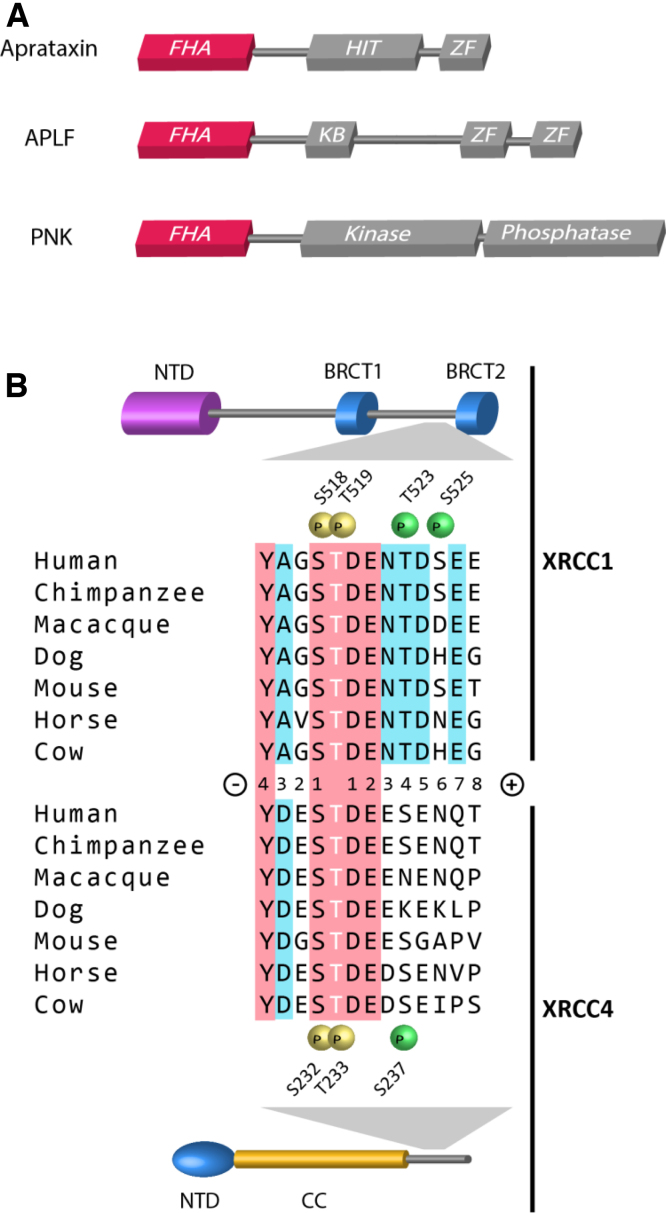
Domain structure and CK2-phosphorylation sites of DNA repair proteins. (A) Schematic representation showing forkhead-associated (FHA), histidine triad (HIT), Ku-binding (KB), zinc finger (ZF), kinase and phosphatase domains of aprataxin, APLF and PNKP. (B) Schematic representation of N-terminal (NTD), BRCA1C-terminal (BRCT) and coiled-coil (CC) domains of XRCC1 and XRCC4 and interspecies sequence conservation of CK2 sites. Positions of CK2-phosphorylation sites in the core motif are denoted with yellow spheres and subsidiary CK2-sites denoted with green spheres. Core motif residues conserved in both XRCC1 and XRCC4 are highlighted in red and residues conserved within XRCC1 or XRCC4 are highlighted in light blue.

Whilst many of the repair pathway components bind to different domains on the scaffolding proteins, three DNA-end modification proteins PNKP, aprataxin and APLF potentially compete for the same binding sites on XRCC1 and XRCC4. PNKP has dual activities; it phosphorylates 5′-OH termini and dephosphorylates 3′-phosphate termini which occur in >50% of breaks induced by oxidative stress [21], [22]. Aprataxin removes AMP from 5′-adenylated DNA which can be formed when DNA ligation is aborted prematurely [23]. The precise role of APLF is yet to be determined although it does possess both endo- and exo-nuclease activity and its depletion is associated with impairment of NHEJ [18]. These three proteins share highly homologous forkhead-associated (FHA) domains (Fig. 1A), which have been shown to function as protein–protein interaction modules through their specific recognition of phosphothreonine-containing motifs on interacting partners [24], [25], [26], [27], [28], [29], [30]. All three bind XRCC1 and XRCC4 in a casein kinase 2 (CK2)-dependent manner [4], [9], [10], [16], [17], [18], [31]. Comparison of CK2 sites in XRCC1 and XRCC4 reveals a common YxxSTDE core motif, in which both serine and threonine are phosphorylated, with subsidiary sites present C-terminal to the core motif (Fig. 1B). Both PNKP and aprataxin FHA domains can bind a triphosphorylated peptide derived from this region of XRCC1 [7], [32] and for PNKP each phosphate has been shown to contribute to binding affinity. Here, we systematically investigate the role of each of the XRCC1 and XRCC4CK2-phosphorylated residues in binding of the aprataxin and APLF FHA domains. The crystal structure of the APLF FHA domain bound to a triphosphosphorylated XRCC4 peptide, together with NMR titration experiments of aprataxin and XRCC1 peptides are used to explore the structural basis for multiple phospho-peptide binding. Together, these data reveal distinct but overlapping binding modes and specificities for this important family of DNA-damage responsive FHA domain proteins that are, in turn, differentially regulated by multi-site phosphorylation of their binding targets.

## 2 Materials and methods

### 2.1 Protein expression and purification

The genes for aprataxin residues 1–105 and APLF residues 1–106 were amplified using primer sets which incorporated 5′ BamHI and 3′ XhoI sites. PCR products and pGEX-6P-1 vector were digested with BamHI and XhoI and religated. All point mutations were generated using a QuickChange Site-Directed Mutagenesis Kit (Stratagene) following the manufacturer's recommended protocol.

For expression, the pGEX-6P-1/aprataxin-FHA constructs were transformed into *Escherichia coli* strain BL21 (DE3) and the pGEX-6P-1/APLF-FHA constructs transformed into the strain Rosetta2 (DE3). Cells were grown in LB at 37 °C to an *A*_600_ of 0.5, induced with 0.4 mM isopropyl 1-thio-β-d-galactopyranoside and incubated at 18 °C for a further 16 h before harvesting. Cells were lysed by sonication and clarified by centrifugation (20,000 × *g* × 30 min). The supernatant was applied to a glutathione-4B resin (Amersham) and cleaved from the resin with rhinovirus 3C protease. Proteins were purified further by gel-filtration chromatography on a Superdex 75 matrix in 20 mM Tris pH 7.5, 150 mM NaCl, 5 mM DTT. Selenomethionine-labelled APLF L91 M protein was expressed in Rosetta2 (DE3) cells cultured in SelenoMet base media and nutrients supplemented with seleno-methionine solution (Molecular Dimensions Ltd.) and purified as the wild-type protein.

### 2.2 Isothermal titration calorimetry

Phosphopeptides based on the XRCC1 sequence 515-YAGSTDENTDSEEHQ-529 and the XRCC4 sequence 229-YDESTDEES-237 were synthesised with amidated C-termini to avoid potential end-effects of a free carboxy-terminus by Dr. W. Mawby (University of Bristol), purified by HPLC and characterised by mass spectrometry. FHA-phosphopeptide binding was quantified by isothermal titration calorimetry using a Microcal Omega VP-ITC calorimeter (MicroCal Inc., Northampton, MA). Protein was dialysed against ITC buffer (50 mM HEPES pH 7.5, 150 mM NaCl, 5 mM β-mercaptoethanol) and peptides were dissolved in the dialysis buffer. Experiments were carried out at 22 °C and involved 30 successive 10 μl injections of peptide solution into a sample cell containing protein solution. Titrations with XRCC1 peptides used peptide at 1 mM and protein at 100 μM and titrations with XRCC4 peptides used peptide at 300 μm and protein at 30 μM. Heats of dilution were subtracted and binding isotherms were plotted and analysed with MicroCal origin version 7.0, assuming a single-site binding model.

### 2.3 Crystallization and structure determination

Selenomethionine-labelled protein and peptide were combined in a 1:3 ratio, with protein at a concentration of 10 mg/ml, in 50 mM HEPES pH 7.5, 150 mM NaCl, 5 mM β-mercaptoethanol. The complex crystallised from hanging drops set up at 18 °C with equal volumes of protein and reservoir solution 0.1 M Tris pH 8.0, 30% w/v PEG 3350, 0.2 M MgCl_2_. Crystals grew within one week and were transferred into cryoprotectant (50 mM Tris pH 8.0, 15% w/v PEG 3350, 0.1 M MgCl_2_, 75 mM NaCl, 25 mM HEPEs pH 7.5) and flash frozen in liquid nitrogen. X-ray diffraction data were collected at a single wavelength of 0.9805 Å on beamline I03 of the Diamond Light Source using an ADSC CCD detector. A total of 720 images were collected with an oscillation angle of 1° and an exposure time of 0.5 s per image. Data were integrated and scaled using DENZO and SCALEPACK [33] and SAD phasing and structure solution carried out by the *Autosol* wizard of the PHENIX software package [34]. Subsequent refinement was carried out at 1.4 Å using the PHENIX refine module of the PHENIX software package and manual model building in Coot [35]. Data collection and refinement statistics are summarised in Table 1. All structure figures were prepared with PyMol (http://www.pymol.org).

### 2.4 NMR spectroscopy

NMR experiments were carried out at 22 °C on Bruker Avance II+ 600 MHz and Avance III 700 MHz spectrometers, each equipped with a cryogenic triple-resonance probe. Protein and peptides were prepared in NMR buffer (20 mM Na Acetate pH 5.8, 50 mM NaCl, 2 mM DTT). Three-dimensional HNCA, HNCO, HNCACB and CBCA(CO)NH experiments were performed to obtain backbone assignments. NMR titration experiments were carried out by adding the unlabelled XRCC1 peptides to ^15^N-labeled aprataxin FHA domain. The initial protein concentration was 92 μM and volumes of 2 mM stock solution of peptide were added until the protein:peptide ratio was 1:4. ^1^H-SOFAST-HMQC [36] spectra were measured after each titration step. All spectra were processed using nmrPipe [37], and analysed using CARA/NEASY [38]. The weighted chemical shift change (in ppm units) of each amide proton (Δ*δ*_HN_) and nitrogen (Δ*δ*_N_) was calculated according to the equation: Δ*δ*_total_ = [(Δ*δ*_HN_*W*_HN_)^2^ + (Δ*δ*_N_*W*_N_)^2^]^1/2^ with *W*_HN_ = 1 and *W*_N_ = 0.154 [39]. Weighted chemical shifts for residues D37, V45, Q46, V61 and V63 were plotted against peptide concentration to determine individual *K*_d_s and averaged to determine the apparent *K*_d_.

## 3 Results

### 3.1 APLF and aprataxin FHA interactions with multi-phospho forms of XRCC1 and XRCC4

**Fig. 2:**
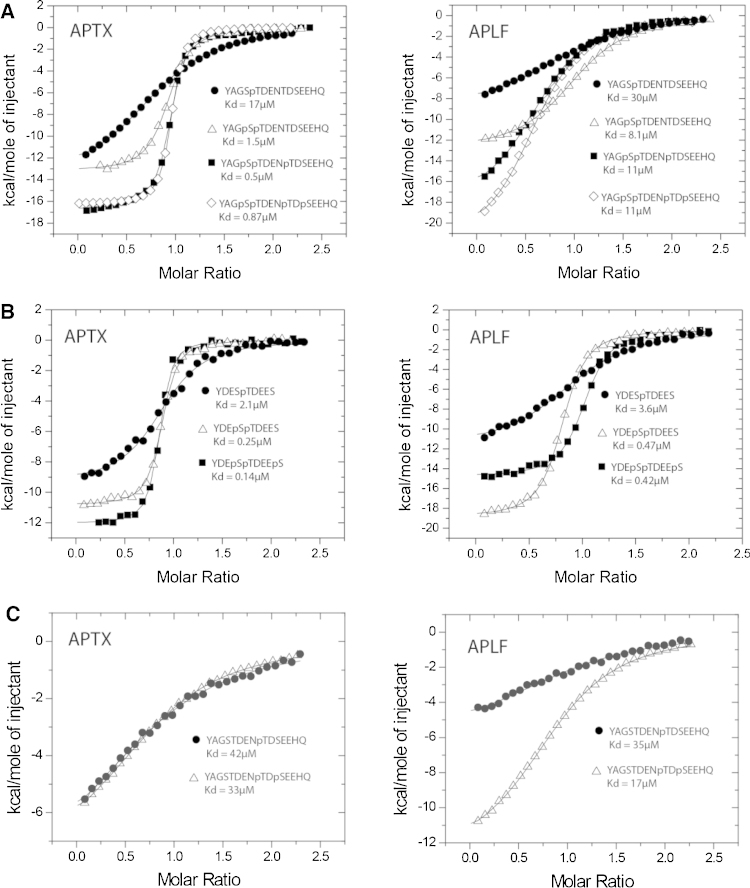
Multiple phosphorylation of XRCC1 and XRCC4 has additive effects on FHA domain binding. Isothermal titration calorimetry of aprataxin (APTX) and APLF binding to peptides phosphorylated on core CK2 XRCC1 sites (A), core CK2 XRCC4 sites (B) and subsidiary CK2 XRCC1 sites (C). Results are representative of experiments performed in triplicate.

APLF and aprataxin bind XRCC1 and XRCC4 following phosphorylation by CK2 on several clustered sites in both proteins. These include the serine and threonine residues within a core heptapeptide sequence, YxxSTDE, found in both XRCC1 and XRCC4, as well as subsidiary sites which lie C-terminal to the core motif and differ between XRCC1 and XRCC4. We used recombinant FHA domains from aprataxin and APLF in isothermal titration calorimetry (ITC) experiments with synthetic phosphopeptides to determine the effects on binding of multiple phosphorylation of XRCC1 and XRCC4 within the core motif (Fig. 2A and B; Tables S1 and S2). APLF and aprataxin can bind both XRCC1 and XRCC4 peptides phosphorylated solely on the threonine in the core motif (XRCC1 Thr519 and XRCC4 Thr233). For both FHA domains, binding to XRCC4 is approximately 8-fold tighter than that to XRCC1. Serine is conserved in the pT −1 position (i.e., one residue N-terminal to the pThr) in both XRCC1 and XRCC4 in all species (Fig. 1B) an additional phosphorylation of this serine improves binding in all cases by factors of between 4- and 11-fold.

A third putative CK2 site is located C-terminal to the core motif in the pT +4 position in both scaffolding proteins; Thr523 in XRCC1 and Ser237 in XRCC4. Additional phosphorylation at XRCC1 Thr523 marginally but reproducibly improves aprataxin binding by ∼3-fold with a smaller effect observed for the addition of pSer237 in XRCC4. In contrast, no significant improvement in affinity is observed for APLF binding to either tri-phosphorylated peptide.

Human XRCC1 contains a fourth predicted CK2-site, Ser525 in the pT +6 position, but phosphorylation of this residue does not appreciably increase binding to either APLF or aprataxin. Of the CK2 sites identified in XRCC1 and XRCC4 outside the core motif, only XRCC1 Thr523 (pT +4) is completely conserved across species (Fig. 1B). In the light of this and our observation that XRCC1 Ser525 and XRCC4 Ser237 make no significant contributions to APLF or aprataxin FHA domain binding, the physiological significance of these two peripheral sites remains unclear.

Since FHA domains specifically recognise phosphothreonine residues, we also tested binding to an XRCC1 peptide phosphorylated solely on the subsidiary Thr523 (Fig. 2C). Interestingly, whereas aprataxin has a clear preference for binding to peptides phosphorylated on the core threonine in XRCC1 rather than the subsidiary one, APLF has approximately equal affinity for both peptides.

### 3.2 Structure of the APLF-XRCC4 complex

In order to determine the structural basis for improved binding of multiply-phosphorylated peptides to aprataxin and APLF, weattempted to co-crystallise these FHA domains with XRCC1 and XRCC4-derived peptides. We were successful in growing well diffracting crystals of the APLF FHA domain with a tri-phosphorylated XRCC4 peptide. Neither the available structures of PNKP FHA domain (PDB: 1UJX, 2BRF) nor that of aprataxin (PDB:3KT9) proved useful for phasing of these X-ray data by molecular replacement but we were able to solve the structure using the SAD method and crystals of selenomethionine-substituted protein and refine against data extending to 1.4 Å resolution (Table 1). The final model contains two complexes each comprising residues 1–104 of APLF with an additional five N-terminal-residues derived from the cleaved GST-tag and the bound tri-phosphorylated XRCC4 phosphopeptide.

**Fig. 3:**
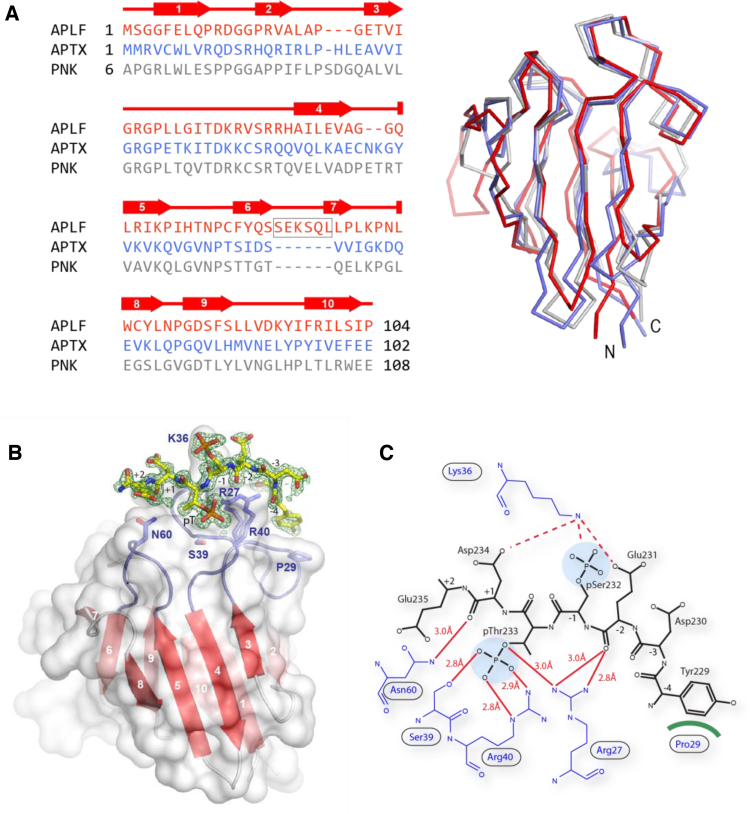
Structure of APLF-FHA:XRCC4 phosphopeptide complex. (A) Sequence alignment and superposition of Cα backbone structures of the APLF FHA domain (red) aprataxin FHA domain (blue) PNKP FHA domain (grey). The positions of β-strands are indicated by arrows above the sequence alignment and the five residue insertion between β6-7 is boxed. (B) Ribbon representation showing the 10 β-strands as red arrows with the molecular surface superimposed. Loops β3–β4 and β5–β6 involved in peptide binding are blue. Seven residues of an XRCC4-derived peptide are shown in stick representation modeled into 2Fo-Fc density. The phosphothreonine binds in the canonical binding pocket whilst phosphoserine protrudes directly away from the FHA domain surface. (C) Schematic representation of protein-peptide contacts between APLF FHA and triphosphorylated XRCC4 peptide. Hydrogen bonds and van der Waals interactions are denoted by red lines and green crescents respectively.

Comparison of the APLF FHA domain structure with those of PNKP [32] and aprataxin [40] shows that, as expected from the relatively high degree of sequence homology, their FHA domain β-sandwich folds are similar overall (Fig. 3A**)**, although, consistent with sequence comparison, the APLF structure is most divergent and contains a six-residue extension in loop β6–β7.

The overall structure of the peptide complex along with electron density for the bound XRCC4 peptide is shown in Fig. 3B. The XRCC4 phosphopeptide adopts an orientation similar to that observed in previously reported FHA/phosphopeptide structures [41], lying across the tips of loops β3–β4 and β5–β6. The core phosphothreonine is secured by a hydrogen-bonding network involving Arg27, Ser39 and Arg40 (Fig. 3C). A second set of hydrogen bonds constrains the peptide backbone on either side; the Arg27 side-chain interacts with the pT −2 and the Asn60 side-chain hydrogen-bonds with the pT +1 carbonyl. Tyr229 in position pT −4 is held in place by a hydrophobic stacking interaction with Pro29. Identical interactions are present in structures of PNKP bound to an XRCC4 peptide [42]. In accordance with the lack of binding effect associated with phosphorylation of Ser237, only residues pT −4 to pT +2 could be modelled into the electron density, indicating that the C-terminal end of the peptide, including Asp236 at pT +3, and pSer237 at the pT +4 position, is mobile.

### 3.3 NMR analysis of the aprataxin binding site

We were not able to obtain crystals of aprataxin FHA in complex with any XRCC1 or XRCC4 phosphopeptide, but were able to use solution NMR to examine its interactions with various phosphorylated XRCC1 peptides. Triple-resonance experiments were used to assign the backbone chemical shifts of aprataxin and HMQC titration experiments with several XRCC1-derived peptides allowed identification of the residues involved in binding by chemical-shift perturbation. This information could then be related to our previously determined X-ray structure of the aprataxin FHA domain [40].

**Fig. 4:**
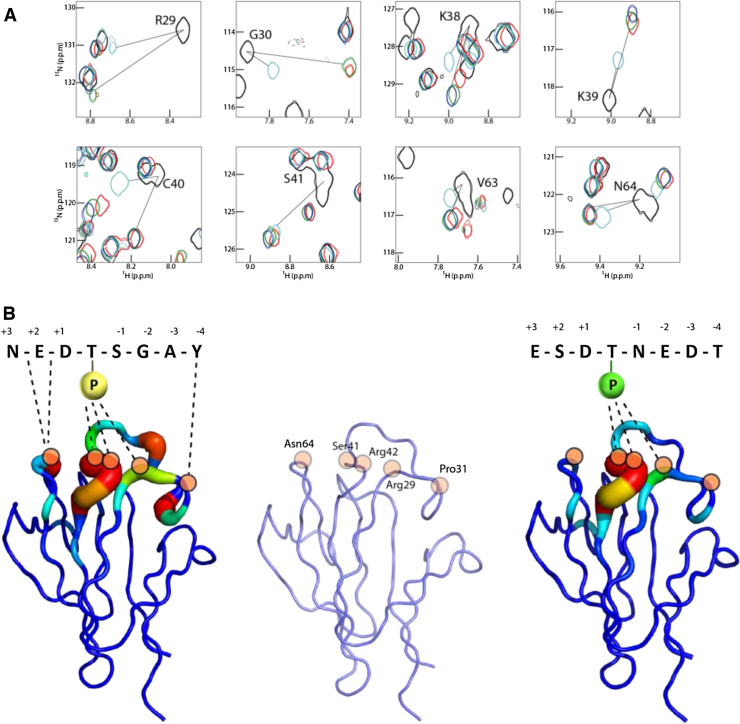
Aprataxin phosphopeptide interactions. (A) Selected chemical shifts from ^1^H [36] -SOFAST-HMQC spectra (22 °C) of 92 μM aprataxin FHA domain, free (black) and in complex in a 1:4 ratio with XRCC1 peptides YAGSpTDENTDSEEHQ (red), YAGpSpTDENTDSEEHQ (green), YAGpSpTDENpTDSEEHQ (dark blue) and YAGSTDENpTDSEEHQ (cyan). Free and the final bound chemical shift positions are connected by black lines. (B) Predicted interactions between aprataxin and XRCC1 peptides phosphorylated on the core-site (yellow sphere) and subsidiary site (green sphere). The FHA trace is colored on a blue to red scale to indicate the degree of chemical shift movements in ^1^H [36] -SOFAST-HMQC titrations.

Spectra for titrations with core site mono- di- and tri-phosphorylated XRCC1 peptides are essentially identical (Fig. 4A) but allow us to estimate overall apparent affinities of ∼7 μM, ∼2 μM and <1.0 μM μM for mono-, di- and tri-phosphorylated peptides, respectively (Table S5). Although the affinity constants for the tighter binding peptides are not well defined due to the high concentration of protein required for the NMR experiment, they are, nonetheless, broadly consistent with the additional contributions of the second and third phosphosites measured by ITC. The majority of residues exhibiting chemical shift changes were within loops β3–β4 and β5–β6 (Fig. 4B). Major shifts of the Arg29 and Ser41 NH resonances, the major pThr-interacting residues, confirmed that the peptide binding mode is similar to that generally observed in other FHA domain systems. In agreement with the APLF/XRCC4 crystal structure, the majority of shifts occur in residues that would be predicted to interact with peptide positions N-terminal to the pThr whilst, NH shifts for aprataxin residues equivalent to those that bind to the specificity-defining peptide pT +3 position in non-aprataxin family FHA domains, are sparse or absent. Interestingly, a similar set of residues was involved in chemical shift changes associated with binding of the peptide solely phosphorylated on the subsidiary threonine (*K*_dapp_ = 7.0 μM), indicating that it binds with the phosphothreonine in the canonical FHA domain phospho-binding pocket rather than at a distinct site.

### 3.4 Structural basis of increased affinity for pSpT forms of XRCC1/4

The XRCC4 peptide contains acidic residues in positions pT −2, pT −3, pT +1 and pT +2. The PNKP/XRCC4 complex suggests that Arg44 provides electrostatic recognition of Glu pT −2 and Asp pT +1 whilst Lys45 may provide additional electrostatic recognition of Asp pT +1 and Glu pT +2 [42]. APLF Arg37, equivalent to PNKP Lys45, does not form any phosphopeptide interactions, instead assuming a structural role through two salt bridges with Asp35 in the β3–β4 loop of the FHA domain. Side-chain density for APLF Lys36, equivalent to PNKP Arg44 is very poor suggesting that it is highly mobile. However, it appears to be in a suitable position for electrostatic recognition of both Glu pT −2, Asp pT +1 and pSer at pT −1 (see below).

**Fig. 5:**
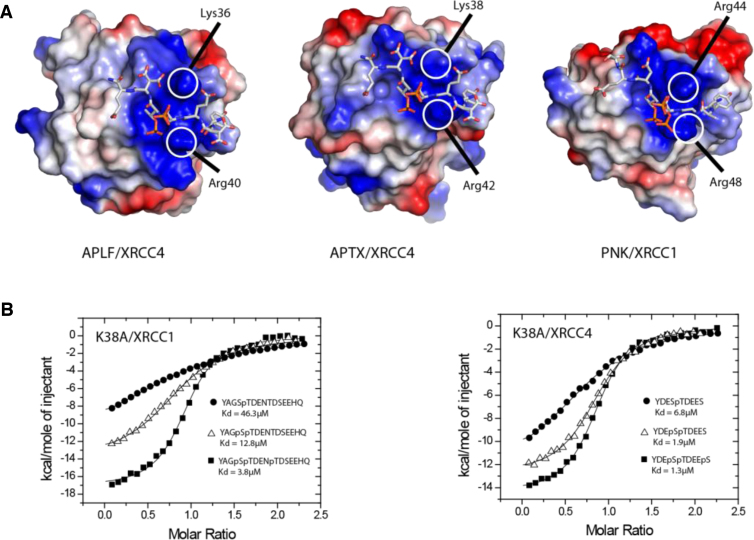
Contribution of basic residues to phosphopeptide binding. (A) Comparison of the electrostatic potential surfaces at the phosphopeptide-binding sites of the APLF, aprataxin (APTX) and PNKP FHA domains. The structure of the aprataxin/XRCC4 complex was modelled on the basis of an overlap of the X-ray structure of the isolated FHA [40] with that of the APLF complex (this study). (B) Effect of aprataxin Lys38 mutation on XRCC1 and XRCC4 phosphopeptide binding. Binding is decreased relative to the wild-type 2.7-, 8.5 and 8.8- fold respectively for mono-, di- and tri-phosphorylated XRCC1 peptides and 3.2-, 7.6- and 9.3-fold respectively for XRCC4 peptides.

The binding studies described above underline clearly the major contribution of the primary pThr site in interactions with both XRCC1 and XRCC4. Of the remaining known phosphorylation sites within these motifs, the absolutely conserved pSer at pT −1 shows the greatest additional contribution to overall affinity of both aprataxin and APLF FHA domains for XRCC1 and XRCC4. Interestingly, in the APLF/XRCC4 structure the pT −1 phosphoserine assumes an upright, solvent exposed conformation, making no direct contacts to FHA domain residues (Fig. 3B**)**. By way of comparison, the structure of PNKP FHA domain bound to a tri-phosphorylated XRCC1 peptide shows two major conformations for the pT −1 phosphoserine of which the lower occupancy conformation resembles that seen in the APLF/XRCC4 complex. A second, higher occupancy conformer is involved in contacts with symmetry-related molecules and calcium ions in the lattice but, nevertheless, makes substantial interactions with Arg48 from the β3 to β4 loop. In our APLF complex, XRCC1 pSer518 is not constrained by crystal contacts but the electron density is also suggestive of additional, low occupancy conformations. Importantly, PNKP Arg48 is conserved in both APLF (Arg40) and aprataxin (Arg42), and APLF Arg40 and PNKP Arg48 adopt identical positions in the respective peptide complex structures (Fig. 5A). Furthermore, from the APLF and PNKP structures of FHA domains bound to multiply phosphorylated peptides, Lys36 in APLF, and its structurally equivalent residues Arg44 in PNKP, and Lys38 in aprataxin appear to be appropriately positioned for interaction with the pSer at the -1 position. Moreover, we have previously shown that mutation of aprataxin Lys38 substantially reduces binding to di-phosphorylated pSDpTD CK2 sites within the DNA-damage mediator protein, Mdc1, where the additional serine phosphorylation occurs at pT −2 [40]. We therefore examined the effects of mutating Arg42 and Lys38 on XRCC1 and XRCC4 peptide binding to aprataxin (Tables S3 and S4 respectively). ITC measurements showed that the aprataxin R42A mutation severely compromised binding to all XRCC1 and XRCC4 peptides, with an interpretable ITC signal only observable for di- and tri-phosphorylated XRCC1. This presumably reflects loss of the substantial interactions with the core pThr. Although these data emphasise the importance of this residue in overall peptide binding, it is clearly impossible to discern any contribution of aprataxin Arg42 to the additional affinity accrued from accessory serine phosphorylation implied by the structural data. However, data from the aprataxin K38A mutant were more illuminating and showed a three-fold decrease in binding to both monophosphorylated XRCC1 and XRCC4 peptides (Fig. 5B). This is likely due to electrostatic interactions with Asp +1 that is present in both XRCC1 and XRCC4, although additional contact with Glu -2 that is only found in XRCC4 may contribute in this particular complex. More importantly, we noted that while the phosphorylation of Ser in the pT −1 position increases wild-type aprataxin binding by ∼11-fold and 8-fold to XRCC1 and XRCC4 respectively, the effect in the context of the aprataxin K38A mutant is significantly reduced in both cases, suggesting that this residue contributes at least part of the increased affinity provided by the accessory Serine ‘-1’ phosphorylation.

## 4 Discussion

PNKP, APLF and aprataxin contain closely related FHA domains, through which they bind to XRCC1 and XRCC4 following phosphorylation by CK2. In addition, we have previously shown that aprataxin associates through its FHA domain with diphosphorylated pSDpTD CK2 motifs in Mdc1 [40] that are also sites of interaction for the FHA and BRCT-repeat domains of the Nbs1 subunit of the Mre11/Rad50/Nbs1 complex [43]. Examination of CK2 sites in XRCC1 and XRCC4 reveals a common core YxxSTDE motif, in which both serine and threonine are canonical CK2 sites, with one or two subsidiary sites C-terminal to this motif. The close proximity of these sites suggests that more than one phosphate may contribute to FHA domain binding. Indeed, studies of the PNKP FHA domain have shown that mono- di- and tri- phosphorylated XRCC1 peptides bind with progressively increasing affinity [32]. Here we have investigated the interactions of XRCC1 and XRCC4 with the two other extant members of this atypical FHA domain protein family, aprataxin and APLF, by a combination of structural and biochemical approaches.

### 4.1 Multi-phosphorylation of XRCC1/4 differentially affects FHA domain affinity and specificity

We have shown that APLF and aprataxin can bind XRCC1 and XRCC4 peptides phosphorylated solely on the core threonine residue. Affinity for XRCC4 is approximately 8-fold higher in both cases and PNKP shows a similar, but less prominent preference [16], [32]. This consistently higher affinity for XRCC4 over XRCC1 is, most likely, due to the presence of additional acidic residues in the pT −1 and pT −2 positions in XRCC4 that are absent in XRCC1 and which can form electrostatic interactions with the positively charged binding surface characteristic of all three FHA domain structures (see below).

As reported for PNKP/XRCC1 interactions [32], additional phosphorylation of Ser at the pT −1 position in the core motif of XRCC1 or XRCC4 improves binding affinity for both APLF and aprataxin. However, the increase in APLF binding to XRCC1 is less (∼3-fold) than for all other combinations (7–11 fold). Furthermore, phosphorylation of threonine in the pT +4 position of XRCC1 somewhat increases binding affinity for aprataxin and PNKP but not for APLF. These combined effects mean that, in their triphosphorylated states, XRCC1 and XRCC4 bind to aprataxin with comparable affinities whereas binding of triphosphorylated XRCC1 to APLF is 26-fold weaker than that of triphosphorylated XRCC4. Indeed, whilst the aprataxin domain affinities for both XRCC1 and XRCC4 are clearly influenced by pT +4 phosphorylation, APLF binding is not. Although APLF has been implicated in repair of both single and double-stranded breaks [10], [18], the significantly greater preference of APLF for XRCC4 (Fig. 1B) suggests that its major biological role is in NHEJ of double-stranded lesions, consistent with recent data [44]. Furthermore, we note that no combination of phosphorylations results in favored binding of APLF for XRCC1 over XRCC4. Thus, APLF shows a minimum preference for XRCC4 (defined as *K*_d(XRCC1)_/*K*_d(XRCC4)_) of ∼2-fold up to a maximum of 26-fold for the triphosphorylated species. In contrast, as with PNKP, aprataxin shows a range of specificities depending on phosphorylation, which vary from a 121-fold discrimination in favor of XRCC4 to a 5-fold preference for tri-phospho XRCC1 over mono-phosphorylated (pT233) XRCC4. Thus, our data suggest a mechanism for CK2-dependent modulation of XRCC1/XRCC4 specificity through patterns of multi-site phosphorylation that decrease the discrimination of aprataxin between these proteins whilst further increasing the preference of APLF for XRCC4.

### 4.2 Surface basicity confers binding versatility

Interactions of APLF, aprataxin and PNKP FHA domains with either XRCC1 or XRCC4 are additionally stabilized by approximately the same degree through phosphorylation of Ser-1. The crystal structure of an APLF/triphosphorylated XRCC4 complex showed this phosphoserine in the core motif to occupy a predominantly upright position facing directly away from the FHA domain surface. This is in contrast to the PNKP/triphosphorylated XRCC1 structure in which the phosphoserine assumes two conformations; one upright and one bent over towards Arg44. NMR titrations of the aprataxin FHA domain with a di-phosphorylated peptide did not identify any interacting residues additional to those implicated in recognition of the mono-phosphorylated peptide. Nonetheless, the greater effect of serine phosphorylation at the -1 position on binding to wild-type compared with the K38A mutant aprataxin suggests a role in phosphoserine recognition. The equivalent residue in APLF, Lys36, is also in a position to make electrostatic interactions with pSer-1, although a lack of density in our structure implies that it is rather mobile. Indeed, it may be that this lack of structural order of aprataxin Lys38, APLF Lys36 and PNKP Arg44 enables recognition of pSer in either the -1 (pSpT; XRCC1/4) or -2 (pSDpTD; Mdc1) positions in different binding partners. This overall versatility appears to be a product of the general distribution of basic side-chains on the FHA domains that are concentrated on the surfaces that interact with the N-terminal residues of the XRCC1/4 binding motifs (Fig. 5). Interestingly, only aprataxin and PNKP have increased affinity for triphosphorylated XRCC1 and XRCC4. This may be attributed to the extended basic surface provided by aprataxin Lys39 and PNKP Lys45 since the equivalent residue in APLF, Arg37, is folded away from the surface. This surface difference may also provide an explanation for observation of differing abilities of these FHA domains to bind poly(ADP-ribose) [45].

### 4.3 Aprataxin, APLF and PNKP assembly on multi-phosphorylated XRCC1

Previous studies of the PNKP FHA domain showed that improved binding affinity observed with increased phosphorylation was associated with the association of two FHA domains with each of core (pS518/pT519) and subsidiary (pT523/pS525) sites within a single XRCC1 peptide [32]. This led to the proposal of a cooperative mechanism in which binding of one FHA domain to the core motif promotes binding at the subsidiary site. Our preliminary ITC observations showed that like PNKP, APLF and aprataxin are also capable of binding an XRCC1 peptide phosphorylated solely at Thr523 within the subsidiary site. Supporting NMR titration data further indicated that this peptide binds with the phosphothreonine in the canonical binding pocket as might be predicted. Nonetheless, an overall conservation of a ‘PNKP-like’ assembly mode is not evident. Firstly, our data indicate that dimerisation of the aprataxin FHA domain does not occur at all and inspection of surface exposed residues on the β10-β11 face of the β-sandwich suggest that Mdc1-like dimerization [46] is not possible (data not shown) Secondly, although we did observe a reproducible increase in stoichiometry for APLF with tri- and tetra-phosphorylated XRCC1 peptides suggestive of 2:1 binding, no indication of co-operativity elicited through FHA–FHA interactions is evident. Indeed, more recent binding experiments employing steady-state fluorescence methods have indicated a 1:1 stoichiometry for binding of PNKP FHA to tetra-phosphorylated XRCC1 [47], more consonant with our APLF and aprataxin data. The reasons for the apparent differences between the two PNKP studies remain unknown and will require further investigation.

## 5 Conclusions

DNA damage generates a variety of different lesion types which each require the action of a particular array of DNA repair proteins. The scaffolding proteins XRCC1 and XRCC4 recruit DNA-repair modulators containing the aprataxin-family FHA domain to the sites of damage through binding to CK2 phosphorylated sites. A complete understanding of the regulated recruitment of aprataxin, APLF and PNKP will require further investigation of the interplay between their respective FHA-mediated interactions and other factors such as the involvement of additional binding domains and patterns of post-translational modification. To this end, we have now shown how the FHA-mediated response of PNKP, aprataxin and APLF to differential phosphorylation of DNA-damage scaffolds such as XRCC1 and XRCC4 plays a major role in determining binding selectivity.

## Conflict of interest

The authors declare that they have no competing interests.

## Accession numbers

Coordinates and structure factors have been deposited with the Protein Data Bank with accession code 5E50.

## Figures and Tables

**Table 1:** Crystallographic statistics.

Data collection	APLF/XRCC4 SeMet
Space group	P 2_1_2_1_2_1_
Cell dimensions	
*a*, *b*, *c* (Å)	38.50 58.54 94.93
*α*, *β*, *γ* (°)	90.0, 90.0, 90.0
Wavelength (Å)	0.98050
Resolution (Å)	30.0–1.38 (1.42–1.38)^a^
*R*_merge_^b^	5.3 (39.9)
*I*/*σ I*	26.7 (3.7)
Completeness (%)	97.8 (80.4)
Redundancy	6.5 (5.1)

Refinement	
Resolution (Å)	28.0–1.38
No. reflections	43,936
*R*_work_^c^/*R*_free_^d^ (%)	13.7/17.4
No. atoms	
Protein	1736
Ligand/ion	147
Water	402
B-factors	
Protein	14.5
Ligand/ion	26.3
Water	33.4
R.m.s deviations	
Bond lengths (Å)	0.011
Bond angles (°)	1.36

a Statistics for outer resolution shell.

b Rmerge = ∑ *_hkl_* ∑ *_i_* | I*_i_* − I |/∑*_hkl_*∑I*_i_* where I*_i_* is the intensity of the ith measurement of a reflection with indexes *hkl* and I is the statistically weighted average reflection intensity.

c *R*-work = ∑||*F*_o_| − |*F*_c_||/∑|*F*_o_| where Fo and Fc are the observed and calculated structure factor amplitudes, respectively.

d *Rfree* is the R-factor calculated with a random 5% of the reflections omitted from refinement.
