# Supplementary material for: Versatility in phospho-dependent molecular recognition of the XRCC1 and XRCC4 DNA-damage scaffolds by aprataxin-family FHA domains
Source: DNA Repair (Amst). 2015 Nov;35:116–25. doi: 10.1016/j.dnarep.2015.10.002 (PMC4655838; doi:10.1016/j.dnarep.2015.10.002)
Supplement: Supplementary file 1 [file mmc1.doc]

**SUPPLEMENTARY MATERIAL**

| **Table S1. ITC binding measurements of wild-type aprataxin FHA domain with XRCC1 and XRCC4 Peptides** | | | | |
| --- | --- | --- | --- | --- |
| **Syringe: Peptide Sequence** | **Kd (µM)** | **N** | **∆H (J mol^-1^)** | **T∆S (J mol^-1^)** |
| **XRCC1**  YAGSpTDENTDSEEHQ | 17 ± 0.4 | 0.85 | -14320 | -7876 |
| YAGpSpTDENTDSEEHQ | 1.5 ± 0.06 | 0.90 | -13620 | -5753 |
| YAGpSpTDENpTDSEEHQ | 0.5 ± 0.05 | 0.94 | -16570 | -8054 |
| YAGpSpTDENpTDpSEEHQ | 0.87 ± 0.05 | 0.92 | -17210 | -9027 |
| YAGSTDENpTDSEEHQ | 42 ± 3.8 | 0.89 | -8119 | -2201 |
| YAGSTDENpTDpSEEHQ | 33 ± 0.87 | 0.87 | -7742 | -1682 |
| **XRCC4**  YDESpTDEES | 2.1 ±0.08 | 0.91 | -9797 | -2138 |
| YDEpSpTDEES | 0.25 ±0.02 | 0.87 | -10990 | -2074 |
| YDEpSpTDEEpS | 0.14 ±0.01 | 0.83 | -12050 | -2785 |
| pT and pS denote phosphothreonine and phosphoserine, respectively. | | | | |

| **Table S2. ITC binding measurements of wild-type APLF FHA domain with XRCC1 and XRCC4 Peptides** | | | | |
| --- | --- | --- | --- | --- |
| **Syringe: Peptide Sequence** | **Kd (µM)** | **N** | **∆H (J mol^-1^)** | **T∆S (J mol^-1^)** |
| **XRCC1**  YAGSpTDENTDSEEHQ | 30 ± 4.2 | 1.01 | -10790 | -4691 |
| YAGpSpTDENTDSEEHQ | 8.1 ± 0.2 | 1.03 | -12850 | -5959 |
| YAGpSpTDENpTDSEEHQ | 11 ± 0.5 | 0.69 | -18050 | -11357 |
| YAGpSpTDENpTDpSEEHQ | 11 ± 0.3 | 0.66 | -22310 | -15635 |
| YAGSTDENpTDSEEHQ | 35 ± 1.1 | 1.04 | -5933 | 80.83 |
| YAGSTDENpTDpSEEHQ | 17 ± 0.5 | 0.91 | -12430 | -5989 |
| **XRCC4**  YDESpTDEES | 3.6 ± 0.3 | 0.98 | -12000 | -4632 |
| YDEpSpTDEES | 0.47 ± 0.02 | 0.80 | -18810 | -10266 |
| YDEpSpTDEEpS | 0.42 ± 0.03 | 1.00 | -14800 | -6915 |
| pT and pS denote phosphothreonine and phosphoserine, respectively. | | | | |

| **Table S3. ITC binding measurements of R42A Mutant of aprataxin FHA domain with XRCC1 and XRCC4 Peptides** | | | | |
| --- | --- | --- | --- | --- |
| **Syringe: Peptide Sequence** | **Kd (µM)** | **N** | **∆H (J mol^-1^)** | **T∆S (J mol^-1^)** |
| **XRCC1**  YAGSpTDENTDSEEHQ | NDB |  |  |  |
| YAGpSpTDENTDSEEHQ | 53.2 ± 5.8 | 0.81 | -6837 | -1062 |
| YAGpSpTDENpTDSEEHQ | 28.7 ± 1.9 | 0.91 | -10390 | -4248 |
| **XRCC4**  YDESpTDEES | NDB |  |  |  |
| YDEpSpTDEES | NDB |  |  |  |
| YDEpSpTDEEpS | NDB |  |  |  |
| NDB indicates no detectable heat change for Aprataxin FHA concentrations of at least  100 µM. pT and pS denote phosphothreonine and phosphoserine, respectively. | | | | |

| **Table S4. ITC binding measurements of K38A Mutant of aprataxin FHA domain with XRCC1 and XRCC4 Peptides** | | | | |
| --- | --- | --- | --- | --- |
| **Syringe: Peptide Sequence** | **Kd (µM)** | **N** | **∆H (J mol^-1^)** | **T∆S (J mol^-1^)** |
| **XRCC1**  YAGSpTDENTDSEEHQ | 46 ± 2.0 | 0.89 | -12690 | -6815 |
| YAGpSpTDENTDSEEHQ | 12.8 ± 0.7 | 0.88 | -14110 | -7493 |
| YAGpSpTDENpTDSEEHQ | 4.4 ± 0.3 | 0.94 | -17580 | -10325 |
| **XRCC4**  YDESpTDEES | 6.8 ± 0.5 | 0.82 | -13750 | -6903 |
| YDEpSpTDEES | 1.9 ± 0.06 | 0.88 | -12880 | -5133 |
| YDEpSpTDEEpS | 1.3 ± 0.05 | 0.87 | -14490 | -6520 |
| NDB indicates no detectable heat change for Aprataxin FHA concentrations of at least  100 µM. pT and pS denote phosphothreonine and phosphoserine, respectively. | | | | |

| **Table S5. NMR titrations of aprataxin/XRCC1 phosphopeptides** | | | | |
| --- | --- | --- | --- | --- |
| **Residue** | **pT519**  **Kd (µM)** | **pS518pT519**  **Kd (µM)** | **pS518pT519pT523**  **Kd (µM)** | **pT523**  **Kd (µM)** |
| Asp37 | 9.6 | 2.0 | 0.1 | 7.6 |
| Val45 | 8.1 | 2.5 | 0.6 | 9.8 |
| Gln46 | 5.3 | 1.7 | 0.1 | 5.9 |
| Val61 | 4.2 | 1.6 | 0.1 | 4.0 |
| Val63 | 6.8 | 2.1 | 0.5 | 7.9 |
| Mean (SEM) | 6.8 (1.9) | 2.0 (0.3) | 0.3 (0.2) | 7.0 (2.0) |
| Titrations were performed by sequential addition of a 2mM peptide stock to 92μM aprataxin in steps of 0.1, 0.2, 0.4, 0.8, 1.5, 3.1 and 6.0 molar ratio aprataxin:peptide | | | | |
